# X chromosome rearrangement associated with premature ovarian insufficiency as diagnosed by molecular cytogenetic methods: a case report and review of the literature

**DOI:** 10.1186/s13039-024-00676-2

**Published:** 2024-04-03

**Authors:** Zhifang Peng, Renqi Yang, Qing Liu, Binbin Chen, Panpan Long

**Affiliations:** Genetic center, Changsha Jiangwan Maternity Hospital, Changsha, 410000 China

**Keywords:** Premature ovarian insufficiency, Chromosome rearrangement, Karyotype analysis, Whole exome sequencing, Case report

## Abstract

**Background:**

Premature ovarian insufficiency (POI) is a clinical condition characterized by ovarian dysfunction in women under 40. The etiology of most POI cases remains unidentified and is believed to be multifactorial, including factors such as autoimmunity, metabolism, infection, and genetics. POI exhibits significant genetic heterogeneity, and it can result from chromosomal abnormalities and monogenic defects.

**Case presentation:**

The study participant, a 33-year-old woman, presented with a history of irregular menstruation that commenced two years ago, progressing to prolonged menstrual episodes and eventual cessation. The participant exhibits a rearrangement of the X chromosome, characterized by heterozygosity duplication on the long arm and heterozygosity deletion on the short arm by whole exome sequencing(WES) combined with cell chromosome detection.

**Conclusions:**

This study expands the spectrum of mutations associated with POI resulting from X chromosomal abnormalities. WES-Copy number variation analysis, in conjunction with chromosome karyotype analysis and other detection techniques, can provide a more comprehensive understanding of the genetic landscape underlying complex single or multi-system diseases.

**Supplementary Information:**

The online version contains supplementary material available at 10.1186/s13039-024-00676-2.

## Background

Premature ovarian insufficiency (POI) is a heterogeneous disorder characterized by impaired ovarian function in women under 40, encompassing a wide range of ovarian dysfunction. It begins with a diminished ovarian reserve and progresses to premature ovarian failure (POF). Clinical manifestations of POI mainly include oligomenorrhea, elevated gonadotropin levels (follicle-stimulating hormone (FSH) > 25U/L), and decreased estrogen levels [1]. POI is a significant cause of female infertility. The etiology of POI is complex and multifaceted, involving genetic, iatrogenic, environmental, and autoimmune factors. Genetic factors contribute to approximately 25–30% of POI cases [2]. X chromosome abnormalities are among the most common genetic causes of inherited POI, with aneuploidy and rearrangements accounting for approximately 10–13% of POI cases [3].

The study participant, a 33-year-old woman, presented with a history of irregular menstruation that commenced two years ago, progressing to prolonged menstrual episodes and eventual cessation. Currently, she relies on medication to sustain her menstrual cycle. Despite actively engaging in unprotected sexual intercourse for over a year, the participant has been unable to achieve pregnancy. A previous evaluation at another medical facility confirmed the diagnosis of POI.

In this study, we report a case of X chromosome rearrangement in a patient with POI and discuss the potential involvement of genes in the pathogenesis of POI. Additionally, we explore the mechanisms underlying this abnormality in POI and evaluate the utility of whole exome sequencing combined with cell chromosome detection as a genetic diagnostic method.

## Case presentation

### Case report

We present the case of a 33-year-old female patient from China who sought fertility treatment at our hospital due to a history of irregular menstruation over the past two years. The patient initially experienced irregular menstrual cycles, which progressively became prolonged and eventually ceased altogether. She reports no menstrual cramps but relies on medication to maintain menstruation. Despite actively trying to conceive for over a year through regular unprotected sexual intercourse, she has been unable to achieve pregnancy, raising concerns of premature ovarian failure. The patient, has remarried and desires another child, has a previous obstetric history, including one pregnancy resulting in a cesarean section delivery in 2009 (G2P1A1). There is no significant family history; upon physical examination, her height is 148.5 cm with a weight of 50.3 kg. A transvaginal ultrasound conducted on December 7, 2020, revealed the following findings: the size of the uterus was approximately 35 * 33 * 41 mm, the left ovary measured 11 * 10 * 10 mm, with no observable follicles, and the right ovary-like structure measured 17 * 12 * 15 mm, without visible follicles. The endometrial thickness was approximately 3.6 mm. Furthermore, ultrasound examinations of the liver, gallbladder, pancreas, spleen, thyroid gland, and bilateral breasts showed no abnormal findings. Reproductive hormone levels were measured as follows, estradiol (E2) <5.0 pg/ml, FSH: 83.73 mIU/ml, luteinizing hormone (LH): 37.83 mIU/ml, progesterone (P): 0.08 ng/ml, prolactin (PRL): 335.2 μIU/ml, anti-Müllerian hormone (AMH).

The patient’s mother experienced menopause at the age of 53.

## Methods

### Karyotype analysis

The patient’s peripheral blood was subjected to routine laboratory methods to detect chromosome abnormalities. G-banding analysis was performed, and 30 metaphase mitoses were examined, with five mitoses analyzed in detail. The International System for Human Cytogenetic Nomenclature (ISCN) served as the diagnostic criteria for the analysis.

### WES

With full consent, the participant’s blood was collected for DNA analysis. Gene analysis was conducted using targeted capture WES of genomic DNA. The DNA underwent fragmentation, repair, amplification, and purification to prepare the sequencing library. A specific capture probe library (Illumina, San Diego, CA, United States) was utilized to capture DNA sequences of the target region, which encompassed all exon regions of approximately 5,000 Online Mendelian Inheritance in Man (OMIM)-related target genes, 30 bp intron regions upstream and downstream of each exon, and known deep intron region variants. WES was performed on the Illumina NovaSeq 6000 platform, and the NextGENe software was employed to align the sequenced reads with the human reference genome (GRCh37). High-frequency variant filtering was applied using population frequency databases (dbSNP, ExAC, and gnomAD) to analyze single nucleotide variations (SNVs)/indels. Pathogenic mutation sites were evaluated by consulting databases such as dbSNP, OMIM, HGMD, ClinVar, and others. Prediction software, including SIFT, Polyphen2, MutationTaster, and FATHMM, were utilized to assess the conservation and pathogenicity of variants. Additionally, for CNV analysis, low-quality sequencing data (average coverage of coding sequence < 3×) was excluded, and the pathogenicity of Copy number variations (CNVs) was assessed by referencing various databases, including DGV, DECIPHER, OMIM, and published literature. The pathogenicity of SNV and CNV variants was classified following the American College of Medical Genetics (ACMG) guidelines [4].

### Variation notes

To annotate variations, we utilized the Efficient Genosome Interpretation System (Egis; Sierra vast-medical, Shanghai, China) and employed GRCh37 as the human reference genome. For CNV analysis, the bpCNV scanning tool within EGIS was utilized. To establish the background library, we calculated the correlation coefficient (*R* > 0.94) based on the average sequencing depth and exon fragment length of both the target samples and reference samples (20 healthy subjects from the same batch). For exons and chromosome CNV calling in ES data, we employed XHMM [5]. The copy number ratio of exon CNV was determined by dividing the target sample’s exon reads per kilobase million (RPKM) mapped reads value by the average RPKM value of the background library sample.

### Kyoto encyclopedia of genes and genomes (KEGG)

We utilized the Orthology-Based Annotation System (KOBAS) version 3.0, an online biological information database for KEGG pathway enrichment analysis.

## Results

### Karyotype analysis

**Fig. 1:**
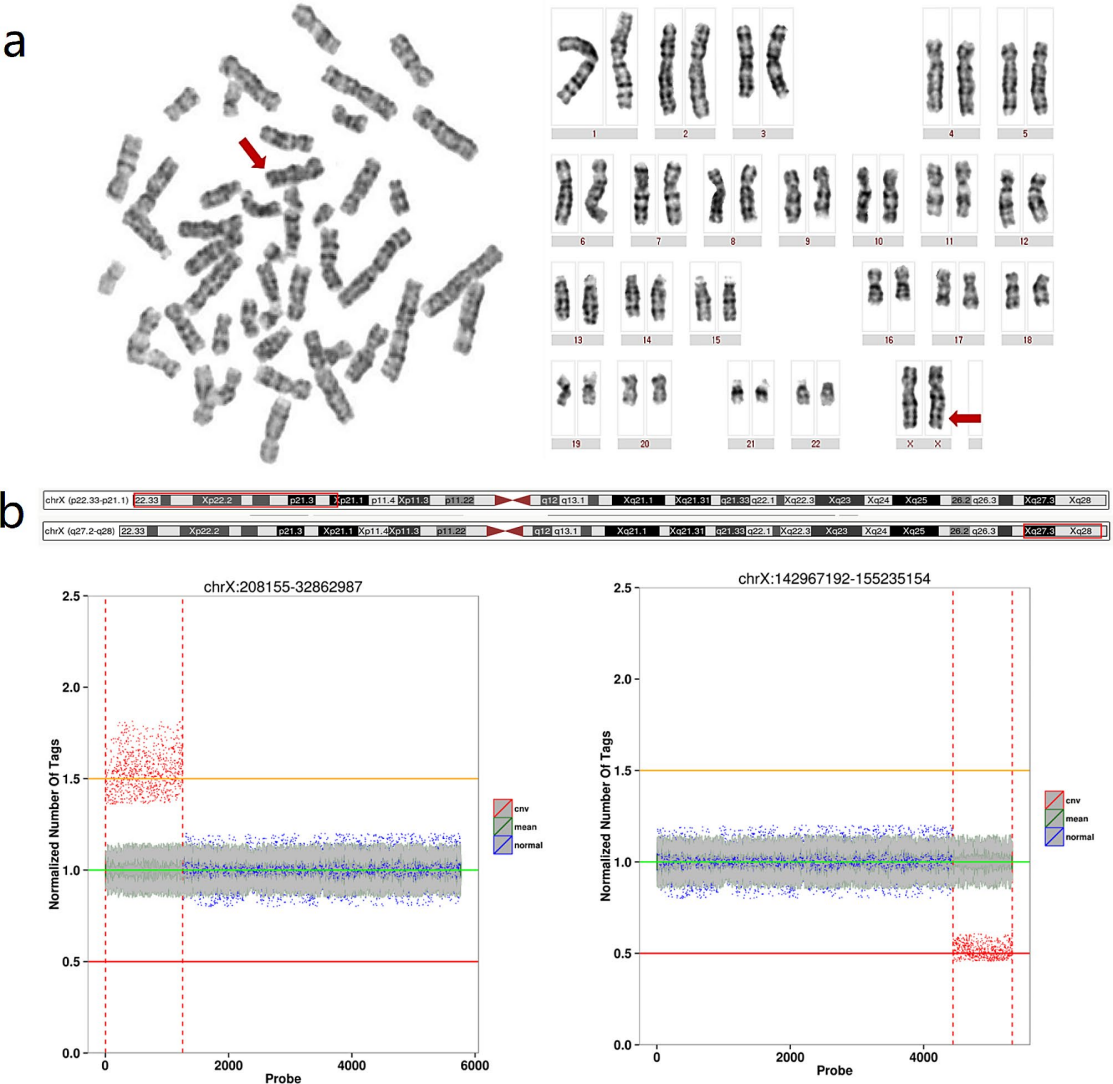
(**a**) Chromosome karyotype analysis involving the examination of 30 metaphase phases and analysis of 5 chromosome karyotypes, yielding an ISCN result of 46, X, der(X)(30), (**b**) Schematic representation of CNV analysis using WES

We examined 30 metaphase phases and analyzed 5 chromosome karyotypes, resulting in an ISCN notation of 46, X, der(X)(30) (Fig. 1a).

### CNV analysis of next-generation sequencing

The CNV analysis of WES data revealed a heterozygous duplication of approximately 32.5 Mb in Xp22.33-p21.1 (chrX: 208155-32862987) and a heterozygous deletion of approximately 12.2 Mb in Xq27.3-q28 (chrX: 142,967,192–155,235,154). The heterozygous repeat region includes 128 OMIM genes, while the heterozygous deletion region includes 113 OMIM genes (Fig. 1b).

### Chromosome-CNV analysis results

The results of chromosome-CNV analysis of WES data are as follows (Table 1). Combining karyotype analysis with CNV analysis, the final ISCN result is 46, X, der(X)(pter→q27.3::p21.1→p22.33::q28→qter).

Reports regarding CNV in these two regions of the X chromosome are associated with recognizable phenotypes. Upon querying related databases, no CNV in these two regions of the X chromosome was found in the Database of Genomic Variants (DGV) database.

**Table 1:** Results of chromosome CNV analysis using WES data

Chromosome number	Chromosome position(Start)	Chromosome position(End)	Area size(kb)	Subband position	CNV type
X	208,155	32,862,987	32654.833	p22.33-p21.1	Heterozygous repetition
X	142,967,192	155,235,154	12267.963	q27.3-q28	Loss of heterozygosity

### DECIPHER database query results

The DECIPHER database query results for GRCh37 are as follows (Table 2), X: 208155-32862987 are related to the patient’s phenotype and X: 142,967,192–155,235,154 are related to the participant’s phenotype.

**Table 2:** Results of DECIPHER database query related to patient phenotype

DECIPHER Patient number	gender	Chromosome position (GRCh37)	size	Geneticmode/ genotype	pathogenicity	phenotype
381,702	46,XX	chrX: 11,091–15,606,375	15.60 Mb	Unknown/heterozygo us repetition	Potentially pathogenic	Ovarian dysfunction, spontaneous abortion
287,181	46,XX	chrX: 150,501,252–155,960,418	5.46 Mb	Unknown/ Loss of heterozygosity	pathogenetic	Ovarian insufficiency
359,210	46,XX	chrX: 140,449,718–155,960,418	15.51 Mb	Unknown/ Loss of heterozygosity	pathogenetic	Ovarian insufficiency
381,702	46,XX	chrX : 152,645,554–156,003,242	3.36 Mb	Unknown/ Loss of heterozygosity	pathogenetic	Ovarian dysfunction, Spontaneous abortion
409,164	46,XX	chrX : 154,892,463–155,331,063	438.6Kb	Unknown/ Loss of heterozygosity	uncertain significance	Hypogonadis m, hearing impairment ovarian insufficiency

### KEGG pathway enrichment analysis

**Fig. 2:**
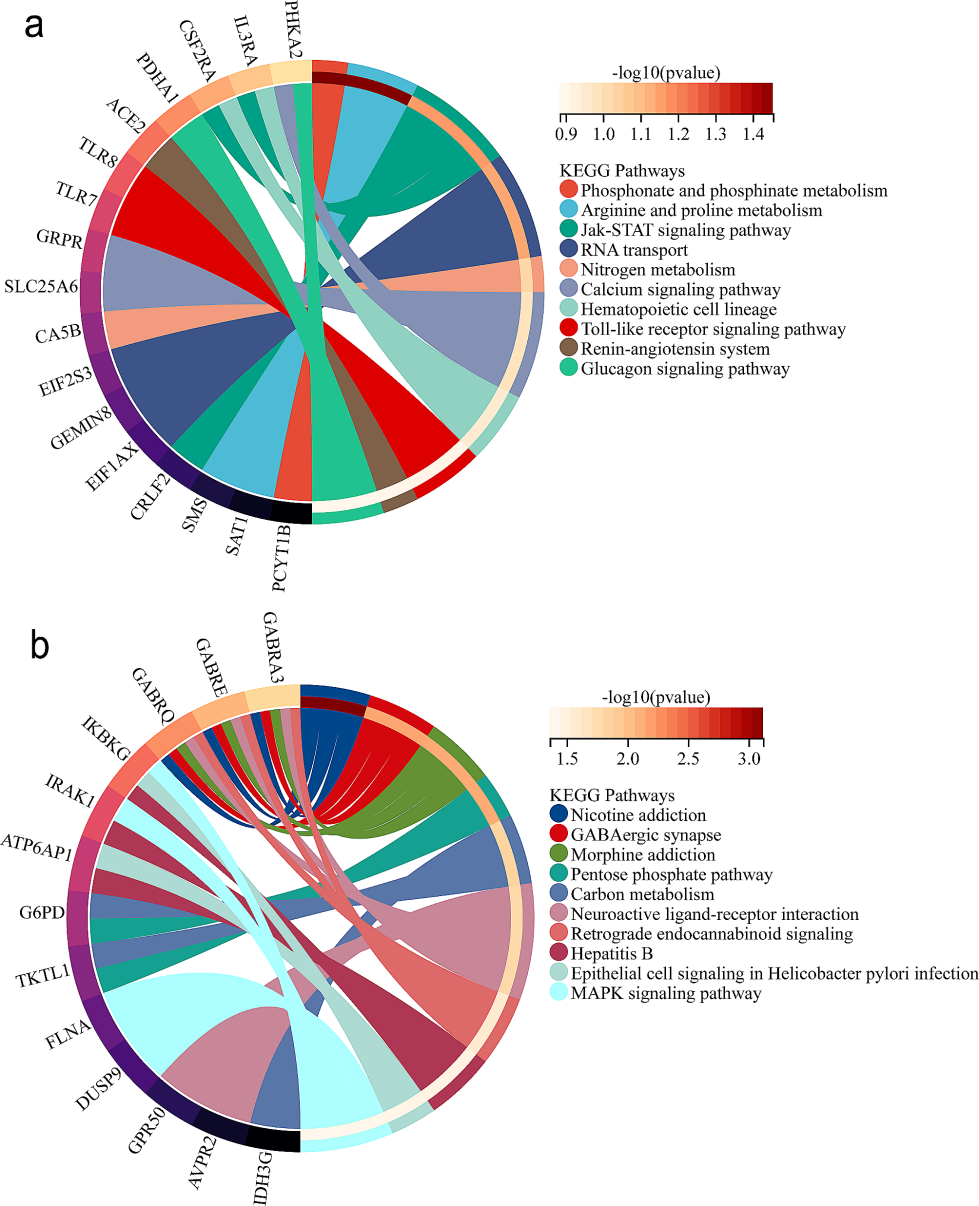
(**a**) KEGG analysis of 128 OMIM genes, (**b**) KEGG analysis of 113 OMIM genes, Each row corresponds to an enriched function, with the length of the bar indicating the enrichment ratio calculated as the “input gene number” divided by the “background gene number.” The bar color corresponds to the color in the circular network above, representing different clusters. For each cluster, if there are more than five terms, the top 5 with the highest enrichment ratio are displayed

The heterozygous duplication region Xp22.33-Xp21.1 (chrX: 208155-32862987) includes 128 OMIM genes (Additional file 1) and is primarily enriched in the DNA replication pathway, apoptosis, and the PI3K-Akt signaling pathway (Fig. 2a). The heterozygous deletion region Xq27.3-Xq28 (chrX: 142,967,192–155,235,154) includes 113 OMIM genes (Additional file 2) and is mainly associated with metabolic pathways, the phosphatidylinositol signaling system, and GABAergic synapse (Fig. 2b).

## Discussion

The decline in ovarian function is a progressive process. In the nonpregnant state, the adult uterus typically measures between 7 and 9 centimeters in length and 4–5 centimeters in width. The ovary of an adult female is approximately 4 cm x 3 cm x 1 cm and has a weight of 5–6 g. Commencing from the ages of 35–40, the ovary starts to diminish in size, and by the age of 50, concomitant with the cessation of menses, it undergoes notable atrophy. The endometrial thickness in post-menopausal women should not exceed 5 mm. Regarding AMH measurements: an AMH level below 1.1 ng/ml indicates a diminished ovarian reserve; an AMH level below 0.2 ng/ml suggests an impending menopause, and post-menopause, AMH is typically undetectable [6]. Therefore, the concept of POF, proposed by the European Society of Human Reproduction and Embryology (ESHRE), encompasses ovarian insufficiency and POF, reflecting the disease’s progression and heterogeneity. POF refers to the end stage of ovarian function deterioration, presenting as primary or secondary amenorrhea caused by gonadal failure before the age of 40 in women. It is accompanied by endocrine abnormalities such as increased gonadotropin levels, decreased estrogen levels, and perimenopausal manifestations such as reproductive organ atrophy [1].

Numerous rearrangements and monosomies of the X chromosome have been identified in association with female infertility. The maintenance of normal ovarian function relies on the presence of two structurally intact X chromosomes. The Xp11.2-Xp22.1 region frequently exhibits X chromosome short-arm loss in patients with POI, indicating its pivotal role in ovarian function [7]. Another region of utmost importance for the POI phenotype spans from Xq13-Xq21 to Xq23-Xq27. Within the Xq13-Xq21 region, epigenetic regulation controls the down-regulation of oocyte expression motifs on autosomal genes [8–11]. Terminal deletions encompassing Xq13 are often associated with primary amenorrhea, breast hypoplasia, and complete ovarian failure [12, 13]. Terminal deletions in Xq25 or Xq26 more commonly lead to POF than primary amenorrhea. Distal deletions in Xq27 or Xq28 are more prevalent than proximal deletions; however, their impact on height and reproductive function is less pronounced [13, 14].

We investigated deletions in regions associated with POI in the patient using the DECIPHER database (Table 2). Our findings revealed that the deletion regions in the four cases from the DECIPHER database coincide with those observed in the patients detailed in this report. Such findings advocate the hypothesis that these deletion regions could be pathogenic and are manifested in patients diagnosed with POI. A potential pathogenic mechanism might stem from the haploinsufficiency of crucial X chromosome genes, leading to cascading effects that impact transcription, translation, splicing, or genome-wide methylation/chromatin modifications [15–18]. An exhaustive analysis underscores a strong association between this deletion region and POI, and the precise pathogenic mechanism or the pivotal candidate genes warrant in-depth research. Research has pinpointed the *FMR1* gene as a recurrent pathogenic factor in POI [19]. The CGG repeat numbers in the 5’ UTR of the *FMR1* gene can be categorized into four groups: normal, intermediate, pre-mutation, and complete mutation [6]. Intriguingly, around 20% of females possessing alleles in the *FMR1* gene’s premutation range (with CGG repeats spanning from 55 to 200) manifest POI [6, 20]. Prior studies have elucidated that females with repeat numbers ranging between 80 and 100 have a heightened risk of developing POI when contrasted with females with repeat numbers either between 59 and 79 or exceeding 100 [21, 22]. Pertinently, in the presented case, the deletion region on the X chromosome encompassed the entirety of the *FMR1* gene.

Approximately 15–20% of women with an *FMR1* mutation will develop POI [23]. In the Caucasian population, *FMR1* mutation accounts for 5% of sporadic POI cases and 10–15% of familial POI cases, prompting the European Society of Human Genetics to recommend routine *FMR1* testing for women with POI [24, 25]. However, the incidence rate among Chinese females is significantly lower than among white females (ranging from 3.3 to 6.7%) [26]. In this case, the breakpoint for the loss of heterozygosity on the short arm of the X chromosome is identified as q27.3-q28, which encompasses the *FMR1* gene and may serve as the underlying cause of POI.

Chromosomal abnormalities represent a prominent etiology of POI. The preservation of female ovarian function relies upon the presence of two structurally normal X chromosomes. Notably, critical genes essential for ovarian development and function are concentrated within key regions of the X chromosome. Insufficient dosage of genes evading X chromosome inactivation in this region, the “positional effect” of rearrangements on neighboring genes, or non-specific disturbances of meiotic homologous chromosome pairing can all culminate in accelerated follicular atresia, serving as the primary pathogenic mechanism underlying POI resulting from X chromosome aberrations. Certain candidate genes associated with POI have been elucidated by identifying the breakpoints of X-autosomal balanced translocations and segmental X chromosome deletions. Nevertheless, it is worth noting that some X-autosomal translocation breakpoints do not encompass any genes or contain minimal coding genes, suggesting that these translocation breakpoints may disrupt gene function through apparent modification effects on autosomal genes translocated to the X chromosome. Critical regions of the rearranged X chromosome suffer disruption, compromising genes and flanking sequences involved in ovarian function, thereby impairing gene function and regulation both upstream and downstream. This phenomenon may be attributed to haploid or interrupted key genes within these regions, non-specific defects in meiotic pairing, and the positional effect exerted by contiguous genes.

Furthermore, structural changes in the spatial configuration of the X chromosome, abnormal topological structures, and mismatches inherent to meiosis can potentially impact the meiotic checkpoint of germ cells, ultimately leading to increased oocyte apoptosis.

WES serves as a common approach for assessing genetic disorders and has proven successful in screening and diagnosing potential genetic causes. CNVs encompass a range of sizes, ranging from a few hundred base pairs to millions of DNA base pairs, involving duplications or deletions. When combined with chromosome karyotype analysis and other detection methods, WES-CNV analysis can offer more comprehensive genetic information for the molecular diagnosis of complex single or multi-system diseases.

## Conclusion

In conclusion, this study expands the spectrum of mutations associated with POI resulting from X chromosomal abnormalities. WES-CNV analysis, in conjunction with chromosome karyotype analysis and other detection techniques, can provide a more comprehensive understanding of the genetic landscape underlying complex single or multi-system diseases.

## Electronic supplementary material

Below is the link to the electronic supplementary material.

Supplementary Material 1

## Data Availability

The data supporting this study’s findings are available upon request from the corresponding author.

## Abbreviations

POI: Premature ovarian insufficiency
WES: whole exome sequencing
CNV: Copy number variation
POF: Premature ovarian failur
FSH: Follicle-stimulating hormone
E2: Estradiol
LH: Luteinizing hormone
P: Progesterone
PRL: Prolactin
AMH: Anti-Müllerian hormone
T: Testosterone
CNVs: Copy number variations
ACMG: American College of Medical Genetics
RPKM: Reads Per Kilobase Million
KEGG: Kyoto Encyclopedia of Genes and Genomes
ISCN: International System for Human Cytogenetic Nomenclature
ESHRE: European Society of Human Reproduction and Embryology

## References

[1] Webber L, Davies M, Anderson R, Bartlett J, Braat D, Cartwright B, Cifkova R, de Keizer-Schrama M, Hogervorst S, Janse E. ESHRE Guideline: management of women with premature ovarian insufficiency. Hum Reprod. 2016;31(5):926–37. 27008889 10.1093/humrep/dew027

[2] Qin Y, Jiao X, Simpson JL, Chen Z-J. Genetics of primary ovarian insufficiency: new developments and opportunities. Hum Reprod Update. 2015;21(6):787–808. 26243799 10.1093/humupd/dmv036 PMC4594617

[3] Cordts EB, Christofolini DM, Dos Santos AA, Bianco B, Barbosa CP. Genetic aspects of premature ovarian failure: a literature review. Arch Gynecol Obstet. 2011;283(3):635–43. 21188402 10.1007/s00404-010-1815-4

[4] Richards S, Aziz N, Bale S, Bick D, Das S, Gastier-Foster J, Grody WW, Hegde M, Lyon E, Spector E, et al. Standards and guidelines for the interpretation of sequence variants: a joint consensus recommendation of the American College of Medical Genetics and Genomics and the Association for Molecular Pathology. Genet Med. 2015;17(5):405–24. 25741868 10.1038/gim.2015.30 PMC4544753

[5] Fromer M, Moran JL, Chambert K, Banks E, Bergen SE, Ruderfer DM, Handsaker RE, McCarroll SA, O’Donovan MC, Owen MJ, et al. Discovery and statistical genotyping of copy-number variation from whole-exome sequencing depth. Am J Hum Genet. 2012;91(4):597–607. 23040492 10.1016/j.ajhg.2012.08.005 PMC3484655

[6] 谢幸 孔北华. 段涛: 妇产科学, 第9版. 人民卫生出版社; 2018.P.7–9,P.24–25,P.353–354.

[7] Pastore LM, Johnson J. The FMR1 gene, infertility, and reproductive decision-making: a review. Front Genet. 2014;5:195. 25071825 10.3389/fgene.2014.00195 PMC4083559

[8] Fortuño C, Labarta E. Genetics of primary ovarian insufficiency: a review. J Assist Reprod Genet. 2014;31(12):1573–85. 25227694 10.1007/s10815-014-0342-9 PMC4250468

[9] Rizzolio F, Bione S, Sala C, Goegan M, Gentile M, Gregato G, Rossi E, Pramparo T, Zuffardi O, Toniolo D. Chromosomal rearrangements in Xq and premature ovarian failure: mapping of 25 new cases and review of the literature. Hum Reprod. 2006;21(6):1477–83. 16497693 10.1093/humrep/dei495

[10] Rizzolio F, Pramparo T, Sala C, Zuffardi O, De Santis L, Rabellotti E, Calzi F, Fusi F, Bellazzi R, Toniolo D. Epigenetic analysis of the critical region I for premature ovarian failure: demonstration of a highly heterochromatic domain on the long arm of the mammalian X chromosome. J Med Genet. 2009;46(9):585–92. 18628312 10.1136/jmg.2007.056093

[11] Rizzolio F, Sala C, Alboresi S, Bione S, Gilli S, Goegan M, Pramparo T, Zuffardi O, Toniolo D. Epigenetic control of the critical region for premature ovarian failure on autosomal genes translocated to the X chromosome: a hypothesis. Hum Genet. 2007;121(3–4):441–50. 17265046 10.1007/s00439-007-0329-z

[12] Sarto GE, Therman E, Patau K. X inactivation in man: a woman with t(Xq–;12q+). Am J Hum Genet. 1973;25(3):262–70. 4704858 PMC1762535

[13] Simpson JL. Genetic and phenotypic heterogeneity in ovarian failure: overview of selected candidate genes. Ann N Y Acad Sci. 2008;1135:146–54. 18574220 10.1196/annals.1429.019

[14] Simpson JL, Rajkovic A. Ovarian differentiation and gonadal failure. Am J Med Genet. 1999;89(4):186–200. 10727994 10.1002/(sici)1096-8628(19991229)89:4<186::aid-ajmg3>3.0.co;2-5

[15] Simpson JL. Gonadal dysgenesis and abnormalities of the human sex chromosomes: current status of phenotypic-karyotypic correlations. Birth Defects Orig Artic Ser. 1975;11(4):23–59. 1098702

[16] Gravholt CH, Viuff M, Just J, Sandahl K, Brun S, van der Velden J, Andersen NH, Skakkebaek A. The changing Face of Turner Syndrome. Endocr Rev. 2023;44(1):33–69. 35695701 10.1210/endrev/bnac016

[17] Zhang X, Hong D, Ma S, Ward T, Ho M, Pattni R, Duren Z, Stankov A, Bade Shrestha S, Hallmayer J, et al. Integrated functional genomic analyses of Klinefelter and Turner syndromes reveal global network effects of altered X chromosome dosage. Proc Natl Acad Sci U S A. 2020;117(9):4864–73. 32071206 10.1073/pnas.1910003117 PMC7060706

[18] Nielsen MM, Trolle C, Vang S, Hornshøj H, Skakkebaek A, Hedegaard J, Nordentoft I, Pedersen JS, Gravholt CH. Epigenetic and transcriptomic consequences of excess X-chromosome material in 47,XXX syndrome-A comparison with Turner syndrome and 46,XX females. Am J Med Genet C Semin Med Genet. 2020;184(2):279–93. 32489015 10.1002/ajmg.c.31799

[19] Johannsen EB, Just J, Viuff MH, Okholm TLH, Pedersen SB, Meyer Lauritsen K, Trolle C, Pedersen MGB, Chang S, Fedder J, et al. Sex chromosome aneuploidies give rise to changes in the circular RNA profile: a circular transcriptome-wide study of Turner and Klinefelter syndrome across different tissues. Front Genet. 2022;13:928874. 35938026 10.3389/fgene.2022.928874 PMC9355307

[20] Barros F, Carvalho F, Barros A, Dória S. Premature ovarian insufficiency: clinical orientations for genetic testing and genetic counseling. Porto Biomed J. 2020;5(3):e62. 33299945 10.1097/j.pbj.0000000000000062 PMC7722400

[21] Allen EG, Grus WE, Narayan S, Espinel W, Sherman SL. Approaches to identify genetic variants that influence the risk for onset of fragile X-associated primary ovarian insufficiency (FXPOI): a preliminary study. Front Genet. 2014;5:260. 25147555 10.3389/fgene.2014.00260 PMC4124461

[22] Allen EG, Sullivan AK, Marcus M, Small C, Dominguez C, Epstein MP, Charen K, He W, Taylor KC, Sherman SL. Examination of reproductive aging milestones among women who carry the FMR1 premutation. Hum Reprod. 2007;22(8):2142–52. 17588953 10.1093/humrep/dem148

[23] Wang B, Wen Q, Ni F, Zhou S, Wang J, Cao Y, Ma X. Analyses of growth differentiation factor 9 (GDF9) and bone morphogenetic protein 15 (BMP15) mutation in Chinese women with premature ovarian failure. Clin Endocrinol (Oxf). 2010;72(1):135–6. 19438907 10.1111/j.1365-2265.2009.03613.x

[24] Wittenberger MD, Hagerman RJ, Sherman SL, McConkie-Rosell A, Welt CK, Rebar RW, Corrigan EC, Simpson JL, Nelson LM. The FMR1 premutation and reproduction. Fertil Steril. 2007;87(3):456–65. 17074338 10.1016/j.fertnstert.2006.09.004

[25] Genetics ESH. Reproduction ESoH, Embryology**: the need for interaction between assisted reproduction technology and genetics: recommendations of the European Societies of Human Genetics and Human Reproduction and Embryology. Hum Reprod. 2006;21(8):1971–3. 16790613 10.1093/humrep/del202

[26] Guo T, Qin Y, Jiao X, Li G, Simpson JL, Chen Z-J. FMR1 premutation is an uncommon explanation for premature ovarian failure in Han Chinese. PLoS ONE. 2014;9(7):e103316. 25050920 10.1371/journal.pone.0103316 PMC4106897

