# Correction: X chromosome rearrangement associated with premature ovarian insufficiency as diagnosed by molecular cytogenetic methods: a case report and review of the literature

**DOI:** 10.1186/s13039-024-00694-0

**Published:** 2024-10-11

**Authors:** Zhifang Peng, Renqi Yang, Qing Liu, Binbin Chen, Panpan Long

**Affiliations:** Genetic center, Changsha Jiangwan Maternity Hospital, Changsha, 410000 China

**Correction: Molecular Cytogenetics (2024) 17:7**

10.1186/s13039-024-00676-2

The authors wish to note the following two errors in the original article along with their respective corrections:

(1) In the Abstract, the authors correct the sentence, “characterized by heterozygosity duplication on the long arm and heterozygosity deletion on the short arm by whole exome sequencing (WES) combined with cell chromosome detection” to “characterized by heterozygosity duplication on the short arm and heterozygosity deletion on the long arm by whole exome sequencing (WES) combined with cell chromosome detection.”

(2) From the discussion, the authors correct the sentence, “In this case, the breakpoint for the loss of heterozygosity on the short arm of the X chromosome is identified as q27.3-q28,” to “In this case, the breakpoint for the loss of heterozygosity on the long arm of the X chromosome is identified as q27.3-q28.”.

